# Supplementary material for: Novel Phototransformable Fluorescent Protein SAASoti with Unique Photochemical Properties
Source: Int J Mol Sci. 2019 Jul 11;20(14):3399. doi: 10.3390/ijms20143399 (PMC6678895; doi:10.3390/ijms20143399)
Supplement: Supplementary file 1 [file ijms-20-03399-s001.pdf]

Supporting Information

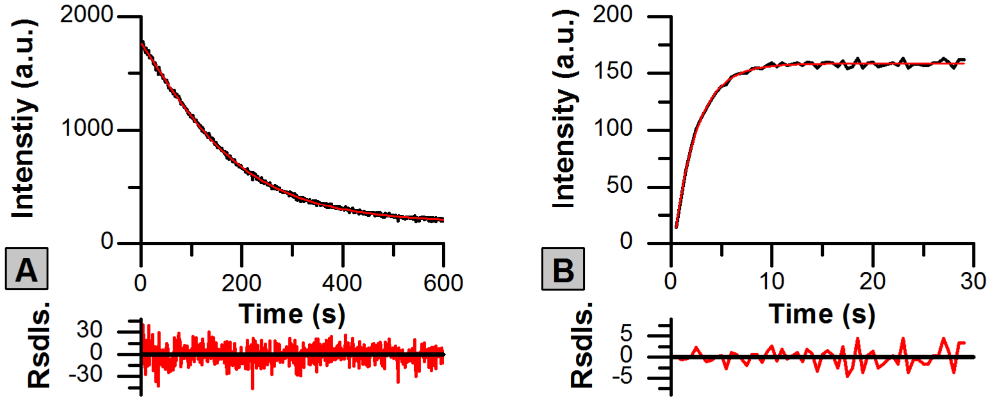

Figure S1. Kinetic analysis of SAASoti green form **A**) 'off' and **B**) 'on' switching curves. 'off'-switching fitted by bi-exponential model, 'on'-switching – by mono-exponential model.

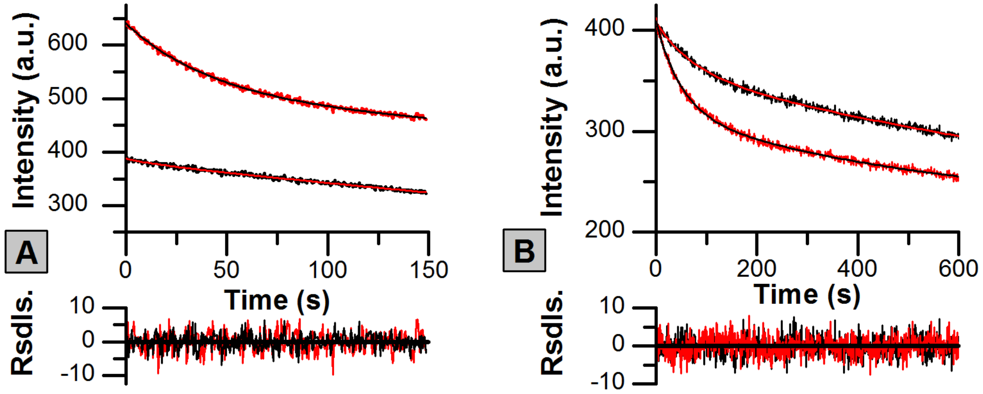

Figure S2. SAASoti red form 'off'-switching kinetic analysis R1 (black lines) and R2 (red lines) fitted by the bi-exponential model. **A**) Fresh sample; **B**) 'Old' sample

Table S1. Peptides V127T SAASoti, in 200 mM $NaHCO_3$

Mass: 25149 **Score**: 167 **Expect**: 1e-016 **Matches**: 11

SAASoti_V127T

Observed	Mr (expt)	Mr (calc)	Ppm	Start	End	Miss	Ions	Peptide
804.4090	803.4017	803.4177	19.97	140	145	0	---	K.TIQWEK.S
932.5124	931.505	931.512	-8.15	139	145	1	--	R.KTIQWEK.
1261.6666	1260.6593	1260.6714	-9.58	140	149	1	---	K.TIQWEKSIEK.M
1385.5983	1384.5911	1384.6048	-9.90	85	95	0	---	K.QSFPEGYSWER.T
1450.7429	1449.7356	1449.7538	-12.53	159	172	0	58	K.GDITMFLLLEGGGK.Y
1648.8334	1647.8262	1647.8119	8.64	71	84	0	---	R.CIVHYPPGIPDYFK.Q
1807.8260	1806.8187	1806.8135	2.91	96	112	0	---	R.TFAFEDGGFCTVSADIK.L
1916.8836	1915.8763	1915.8660	5.36	202	218	0	---	R.TNDDGTQFELNEHAVAR.L
1939.9321	1938.9248	1938.9258	-0.48	186	201	0	---	K.VVEMPQSHYVEHSIER.T
2068.0144	2067.0072	2067.0207	-6.57	185	201	1	---	K.KVVEMPQSHYVEHSIER.T
2963.3391	2962.3318	2962.3677	-12.11	113	138	1	---	K.LKDNCFIHTSMFHGTNFPADGPVMQR.K

Table S2. Peptides V127T SAASoti in 200 mM $NaHCO_3$ after 10 min exposure to 470 nm light (10 W/cm^2)

Mass: 25149 **Score**: 559 **Expect**: 6.3e-056 **Matches**: 14

SAASoti_V127T

Observed	Mr (expt)	Mr (calc)	Ppm	Start	End	Miss	Ions	Peptide
804.4227	803.4154	803.4177	-2.88	140	145	0	---	K.TIQWEK.S
932.5009	931.4936	931.5127	-0.45	139	145	1	---	R.KTIQWEK.S
1261.6565	1260.6492	1260.6714	-7.56	140	149	1	---	K.TIQWEKSIEK.M
1385.5942	1384.5869	1384.6048	-2.88	85	95	0	---	K.QSFPEGYSWER.T
1450.7342	1449.7269	1449.7538	-8.50	159	172	0	---	K.GDITMFLLLEGGGK.Y
1466.7388	1465.7315	1465.7487	-1.72	159	172	0	83	K.GDITMFLLLEGGGK.Y **+ Oxidation (M)**
1648.8134	1647.8061	1647.8119	-3.53	71	84	0	47	R.CIVHYPPGIPDYFK.Q
1807.8217	1806.8145	1806.8135	0.55	96	112	0	144	R.TFAFEDGGFCTVSADIK.L
1916.8804	1915.8731	1915.8660	3.68	202	218	0	---	R.TNDDGTQFELNEHAVAR.L
1939.9325	1938.9253	1938.9258	-0.27	186	201	0	---	K.VVEMPQSHYVEHSIER.T

2068.0275	2067.0202	2067.0207	-0.27	185	201	1	---	K.KVVEMPQSHYVEHSIER.T
2397.2322	2396.2249	2396.2331	-3.45	150	172	1	146	K.MTVSDGIVKGDITMFLLLEGGGK.Y **+ Oxidation (M)**
2963.3518	2962.3445	2962.3677	-7.81	113	138	1	---	K.LKDNCFIHTSMFHGTNFPADGPVMQR.K
3314.4810	3313.4738	3313.5246	-15.35	6	34	0	---	K.QYIPDDMELIFHMDGCVNGHYFTIVATGK.A

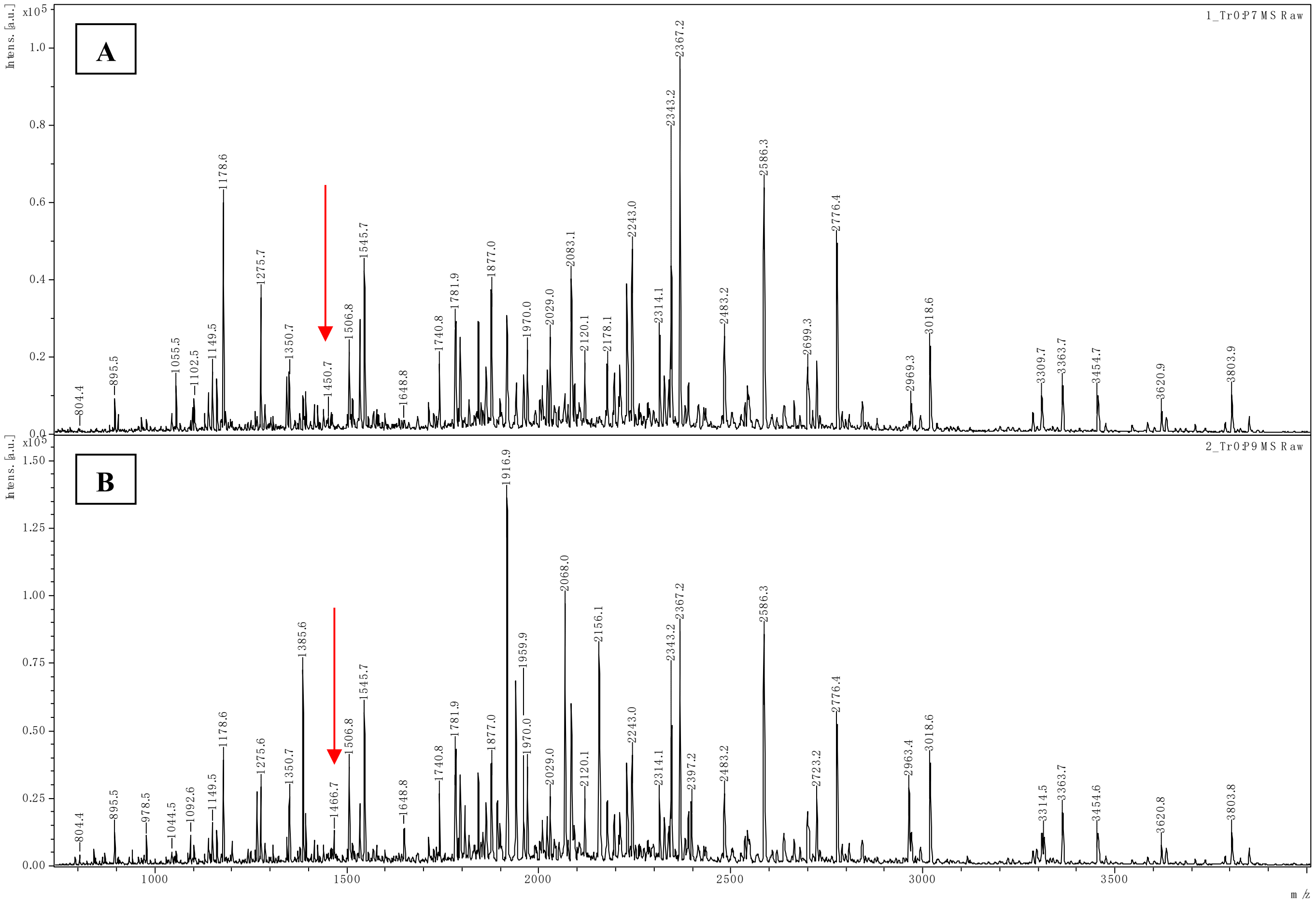

Figure S3. MALDI-TOF/TOF MS Spectra of SAASoti **before** (**A**) and **after (B)** 10 min exposure to 470 nm light (160 mW/cm^2).

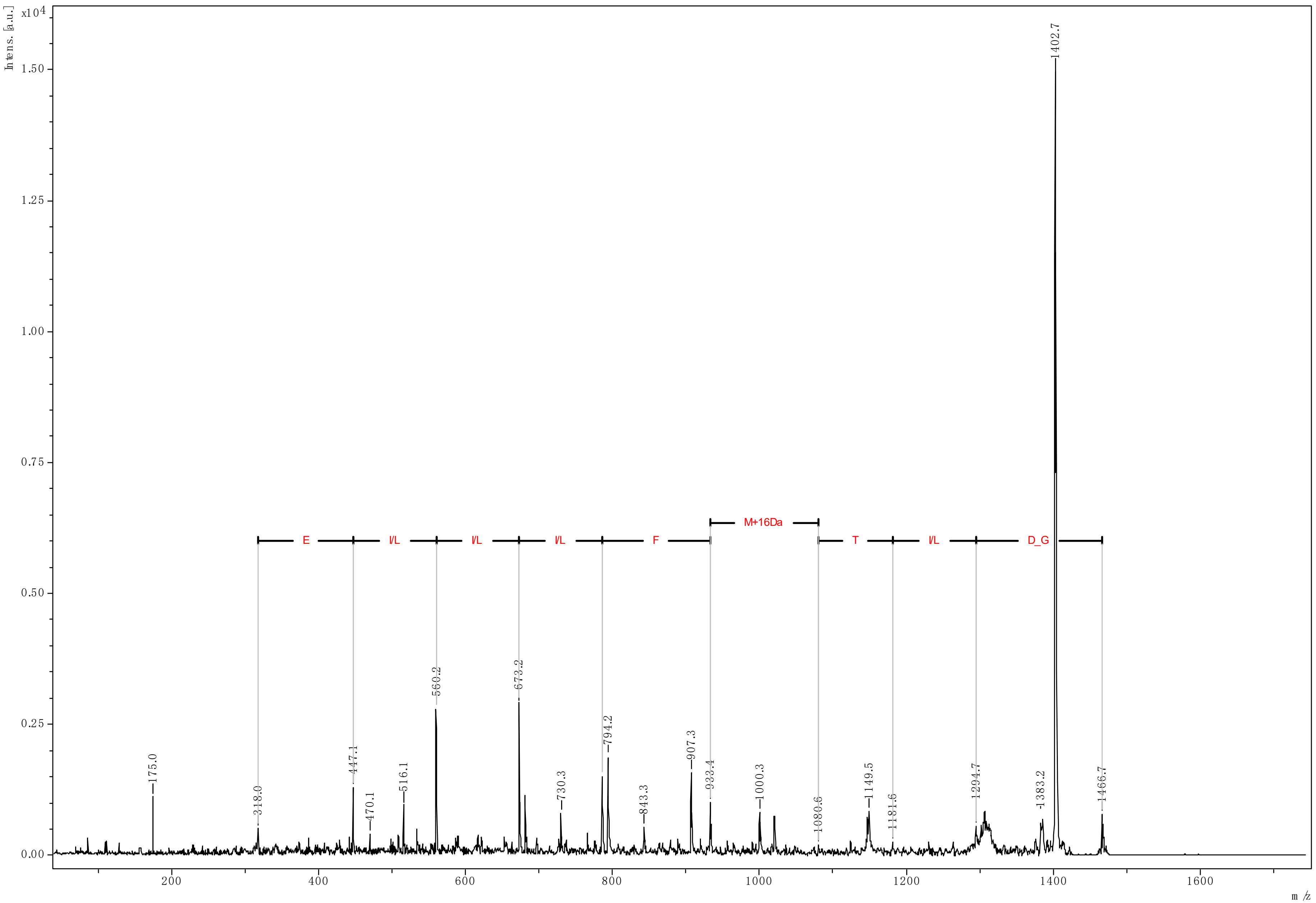

Figure S4. MALDI-TOF/TOF MS/MS spectrum of the molecular ion at m/z 1466.7. M + 16 Da corresponds to M164 oxidation in succinylation in -GDITMFLLLEGGGK- tryptic peptide.
